# Disease aggressiveness signatures of amyotrophic lateral sclerosis in white matter tracts revealed by the D50 disease progression model

**DOI:** 10.1002/hbm.25258

**Published:** 2020-10-26

**Authors:** Robert Steinbach, Nayana Gaur, Annekathrin Roediger, Thomas E. Mayer, Otto W. Witte, Tino Prell, Julian Grosskreutz

**Affiliations:** ^1^ Hans Berger Department of Neurology Jena University Hospital Jena Germany; ^2^ Center for Healthy Ageing Jena University Hospital Jena Germany; ^3^ Department of Neuroradiology Jena University Hospital Jena Germany

**Keywords:** amyotrophic lateral sclerosis, D50 model, diffusion tensor imaging, disease aggressiveness, disease progression, TBSS

## Abstract

Numerous neuroimaging studies in amyotrophic lateral sclerosis (ALS) have reported links between structural changes and clinical data; however phenotypic and disease course heterogeneity have occluded robust associations. The present study used the novel D50 model, which distinguishes between disease accumulation and aggressiveness, to probe correlations with measures of diffusion tensor imaging (DTI). DTI scans of 145 ALS patients and 69 controls were analyzed using tract‐based‐spatial‐statistics of fractional anisotropy (FA), mean‐ (MD), radial (RD), and axial diffusivity (AD) maps. Intergroup contrasts were calculated between patients and controls, and between ALS subgroups: based on (a) the individual disease covered (Phase I vs. II) or b) patients' disease aggressiveness (D50 value). Regression analyses were used to probe correlations with model‐derived parameters. Case–control comparisons revealed widespread ALS‐related white matter pathology with decreased FA and increased MD/RD. These affected pathways showed also correlations with the accumulated disease for increased MD/RD, driven by the subgroup of Phase I patients. No significant differences were noted between patients in Phase I and II for any of the contrasts. Patients with high disease aggressiveness (D50 < 30 months) displayed increased AD/MD in bifrontal and biparietal pathways, which was corroborated by significant voxel‐wise regressions with D50. Application of the D50 model revealed associations between DTI measures and ALS pathology in Phase I, representing individual disease accumulation early in disease. Patients' overall disease aggressiveness correlated robustly with the extent of DTI changes. We recommend the D50 model for studies developing/validating neuroimaging or other biomarkers for ALS.

## Graphical abstract

In the current study, we applied the novel D50 progression model to link clinical data with structural changes assessed using diffusion tensor imaging (DTI) in a large and representative cohort of patients with amyotrophic laterals sclerosis. Doing so, we were able to discover stable associations between neuroimaging indices and clinical parameters that have been previously occluded by the characteristic heterogeneity of individual disease courses. We demonstrate that DTI analyses are particularly sensitive towards disease accumulation‐driven changes in very early disease and in addition that patients' overall disease aggressiveness correlates well with the extend of DTI changes.

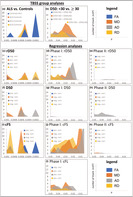

**Abbreviations**
AD: axial diffusivity
ALS: amyotrophic lateral sclerosis
ALSFRS‐R: ALS Functional Rating Scale (Revised)
cFL: calculated functional loss
cFS: calculated functional state
CST: corticospinal tract
DTI: diffusion tensor imaging
FA: fractional anisotropy
FSL: FMRIB Software Library
FWE: family‐wise error
MD: mean diffusivity
MRI: magnetic resonance imaging
PR: progression rate
RD: radial diffusivity
rD50: relative D50
TBSS: tract‐based spatial statistics
TFCE: threshold‐free cluster enhancement
WM: white matter

## 1 INTRODUCTION

Amyotrophic lateral sclerosis (ALS) is a fatal neurodegenerative disease predominantly characterized by the loss of motor‐neuron function; however, given its well documented‐effects on extra‐motor domains of the central nervous system, it is now considered a multisystem disease. Prognoses for this condition remain poor; the median survival is 3 years after symptom onset (Dorst, Ludolph, & Huebers, 2018; Kiernan et al., 2011). Despite considerable ongoing efforts to develop curative therapies, Riluzole is still the only globally approved disease‐modifying therapy (Miller, Mitchell, Lyon, & Moore, 2007; Petrov, Mansfield, Moussy, & Hermine, 2017). In principle, ALS care and research face one major challenge: the disease is characterized by substantial intraindividual and interindividual phenotypic heterogeneity, with highly variable progression and survival rates (Simon et al., 2014; Westeneng et al., 2018).

Reliable biomarkers that can capture patients' individual disease aggressiveness are therefore urgently needed. Magnetic resonance imaging (MRI) has already been recognized as a promising noninvasive technique that provides insights into the neurodegenerative mechanisms underlying ALS (Filippi et al., 2015; Steinbach, Gaur, Stubendorff, Witte, & Grosskreutz, 2018; Turner et al., 2011). In particular, the use of diffusion tensor imaging (DTI) for capturing ALS associated‐pathology in white‐matter (WM) fibertracts and classifying different disease subtypes has been established (Filippi et al., 2015; Muller et al., 2016). Several DTI‐based studies have demonstrated ALS‐associated loss of WM structural integrity on a case–control‐level (e.g., in the corticospinal tract [CST] and corpus callosum), albeit with inconsistent and controversial results, for example, regarding the extent and significance of affected regions (Bede & Hardiman, 2014; Floeter, Danielian, Braun, & Wu, 2018; Li et al., 2012; Zhang et al., 2018). Most important, former DTI studies frequently failed to show correlations of derived structural abnormalities with patients' clinical disease severity (Canu et al., 2011; Geraldo et al., 2018; Keller et al., 2011; Rose et al., 2012; Senda et al., 2011). Within the ALS and wider neurodegenerative research community, this discrepancy is often referred to as the “correlation gap” (Verstraete et al., 2015).

It has been postulated, that longitudinal MRI data would offer the best insight into neuroimaging alterations across the disease course, thus having the potential to close this gap. However, the nature of ALS causes relevant obstacles for serial MRI assessments during patients' course of the disease, for example, due to cognitive impairment, bulbar and respiratory symptoms and poor longevity (Chio et al., 2014; Filippi et al., 2015). It is therefore not surprising, that former longitudinal studies captured rather small sample sizes with assumable selection bias and/or short disease periods with often not more than two to three time points (Agosta et al., 2009; Alruwaili et al., 2019; Bede & Hardiman, 2018; Keil et al., 2012; Menke, Proudfoot, Talbot, & Turner, 2018; Steinbach et al., 2015; van der Graaff et al., 2011). Alternatively, large‐scale cross‐sectional datasets can provide valuable pseudo‐longitudinal substitutes, but require a quantitative understanding of individual disease state and trajectory at the time of MRI acquisition.

The recently developed D50 model provides a novel way to quantify disease aggressiveness separately from disease accumulation even in highly heterogenous ALS cohorts. Briefly, the model incorporates a sigmoidal decline of the ALS Functional Rating Scale (ALSFRS‐R) throughout the disease course because a curvilinear progression was suggested before (Franchignoni, Mora, Giordano, Volanti, & Chio, 2013; Gordon et al., 2010; Senda et al., 2017). The D50 model addresses the difficulty in characterizing functional loss at the individual and population level using traditional clinical indices like the progression rate (PR) (Poesen et al., 2017; Prell, Gaur, Steinbach, Witte, & Grosskreutz, 2020; Steinbach et al., 2020). As such, the model quantitatively (a) captures phenotypic complexity, (b) reduces noise associated with traditional disease progression parameters, and (c) provides distinct descriptors of patients' aggressivity and individual disease covered.

We hypothesize that these parameters close the correlation gap between clinical characteristics of ALS and in vivo measures of cerebral structural integrity. The present study applied tract‐based spatial statistics (TBSS) in a large cohort of patients with detailed D50 model‐based descriptors of the ALS disease process to quantify the tract damage ALS causes depending on disease aggressiveness and phases of individual disease covered.

## 2 METHODS

### 2.1 Subjects

This study was approved by the local Ethics committee (No. 3633‐11/12) and all experimental procedures were performed in accordance with the 1964 Declaration of Helsinki; written informed consent was obtained from individual participants prior to enrolment.

All participants were consecutively recruited from the Department of Neurology at Jena University Hospital. Available neuroimaging of participants, based on a harmonized MRI protocol, were inspected (FLAIR and T1 weighted structural images) by a trained analyst (R. S.) and neuroradiologist (T. M.) and excluded if any relevant intracranial pathologies (e.g., tumors, cysts, stroke, or bleedings) were present. All patients with ALS were examined by a trained neurologist at enrollment and follow‐up visits (minimum of two assessments for each patient) and met the criteria of definite, probable or laboratory‐supported probable ALS (Brooks, Miller, Swash, Munsat,, & World Federation of Neurology Research Group on Motor Neuron, 2000). Exclusion criteria included the presence of (a) juvenile ALS, (b) primary lateral sclerosis, (c) clinically relevant dementia symptoms, (d) any comorbidities that could affect motor performance and (e) a D50 value above 100 months. Finally, 145 patients and 69 healthy controls were included in the analyses (for a CONSORT diagram refer to Supplementary Figure S1).

### 2.2 The D50 disease progression model

**FIGURE 1:**
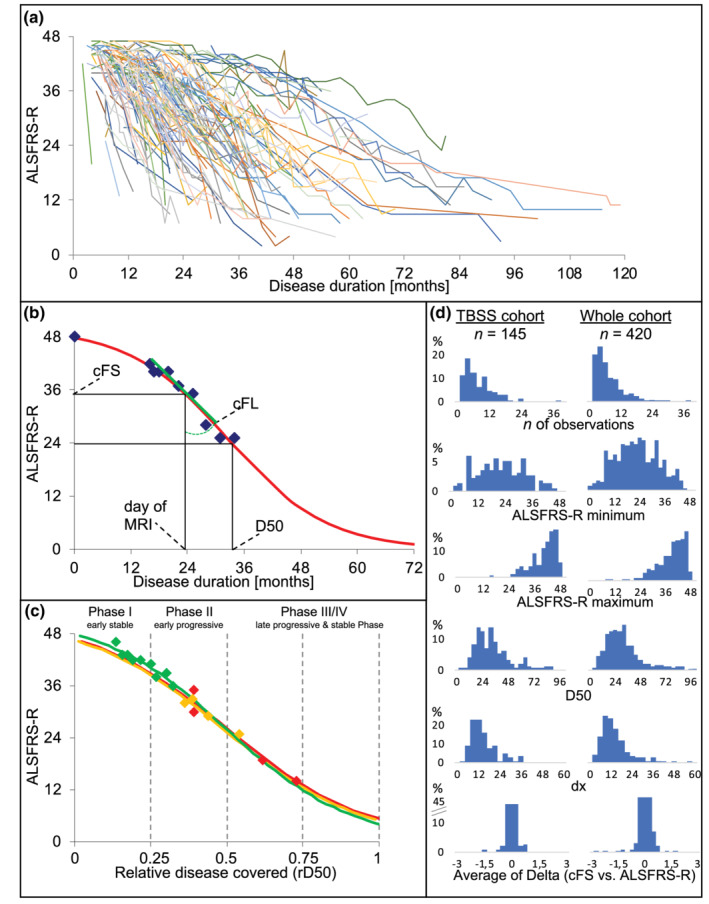
Principles of the D50 disease progression model. (a) The plot shows the temporal decay of ALSFRS‐R scores for all 145 ALS patients. Thus, it illustrates the vastly differing timescales of functional loss between patients. (b) Representative simulated disease curve of a patient with ALS. The sigmoidal disease course (red curve) is modeled based on regularly obtained ALSFRS‐R scores (blue squares) throughout the disease. Resulting parameters: D50 = calculated time point when ALSFRS‐R drops to 24 (50% functionality), cFS = calculated functional state and cFL = calculated functional loss at the time point of examination (here at MRI acquisition). (c) Normalization with rD50, which describes individual disease course covered in reference to D50, allows for comparability between patients who despite vastly different disease aggressiveness profiles all proceed through similar Phases (I–IV) of functional decline. The curves illustrate three representative patients with ALS, either having high (depicted in red, D50 = 9 months), moderate (depicted in yellow, D50 = 19 months) or low (depicted in green, D50 = 84 months) disease aggressiveness. (d) The distributions of variables for the present study‐cohort (histograms in left column) were similar with those for all ALS patient data available at our neuromuscular center (histograms in right column). From top to bottom, the number of obtained ALSFRS‐R scores per patient, the minimum ALSFRS‐R score, the maximum ALSFRS‐R score, and the calculated D50 and dx values are presented. The bottom last row depicts the averaged difference of calculated functionality (cFS) relative to actual ALSFRS‐R scores available per patient. ALS, amyotrophic lateral sclerosis; ALSFRS‐R, ALS Functional Rating Scale (Revised); D50 estimated time in months for an individual to lose 50% of functionality; cFL calculated functional loss, cFS calculated functional state, rD50 relative D50

The D50 model provides parameters of overall disease aggressiveness, local disease activity, and individual disease covered (Poesen et al., 2017;Prell et al., 2020; Steinbach et al., 2020). Figure 1 provides a pictorial overview of how the model estimation was performed based on all the regularly collected ALSFRS‐R scores that were available per patient (Figure 1a). Briefly, the D50 model describes the disease course of individual ALS patients as a sigmoidal state transition from full health to functional loss. The curve is calculated using iterative fitting of available ALSFRS‐R scores (Figure 1b). The value dx describes the time constant of ALSFRS‐R decline and the value D50 is defined as the estimated time taken in months for a patient to lose 50% of his/her functionality (equivalent to an ALSFRS‐R score of 24). Given that dx and D50 correlate linearly in ALS cohorts (Poesen et al., 2017; Prell et al., 2020), the D50 value provides a unified descriptive measure of individual patients' overall disease aggressiveness. This allowed us to classify patients as having either low (D50 ≥ 30 months) or high (D50 < 30 months) disease aggressiveness. The cutoff value of 30 months corresponds to the median of D50 values (here: 28.8 months), that is typically observed in comparable cohorts of patients with ALS treated at our center (Prell et al., 2020; Steinbach et al., 2020). We excluded patients with a D50 value above 100 months and only one ALSFRS‐R score, to ensure a high level of reliability for the calculation of the D50 model.

Normalizing an individual's real‐time disease trajectory to D50 yields the parameter relative D50 (rD50), an open‐ended linear reference scale where 0 signifies symptom onset and 0.5 indicates the time point of halved functionality. Patients can be categorized into at least 3 phases: an early semistable Phase I (0 ≤ rD50 < 0.25), an early progressive Phase II (0.25 ≤ rD50 < 0.5), and late progressive and late stable Phases III/IV (rD50 ≥ 0.5; Figure 1c).

The model also yields two descriptors of local disease activity that can be calculated at any given time point across the patient's disease course (here at MRI); namely the calculated functional state (cFS) and the calculated functional loss (cFL) (Figure 1b). These provide a measure of functional deterioration which greatly reduce the noise inherent to the ALSFRS‐R score (Bakker et al., 2020) and provide adjacent averaging. This was particularly useful in 41 patients who did not receive ALSFRS‐R scoring at the time of MRI. The other 114 patients received an ALSFRS‐R assessment within 10 days prior to or after MRI acquisition (see Supplementary Table S1 for the clinical data of this subcohort, comparing traditional, and D50 disease metrics). Thus, the D50 model enabled the inclusion of all ALS patients in the subsequent neuroimaging analyses.

Figure 1d presents histograms of relevant variables used for the calculation of the D50 model and resulting parameters. The distributions of these variables indicate that the ALS cohort of this study (*n* = 145) well represents the regional ALS population available at our center (*n* = 420).

### 2.3 MRI data acquisition and processing of DTI images

DTI images were obtained in a gradient‐weighted spin‐echo sequence on a 1.5 Tesla Siemens Sonata scanner acquiring 192 sagittal slices with the following parameter settings: repetition time 7,800 ms, echo time 97 ms, flip angle 90°, slice thickness 2.5 mm, pixel size 1.25 mm × 1.25 mm, acquisition matrix 96 × 96. Each scan included both (a) 30 images acquired along 30 noncollinear directions at *b* = 1,000 s/mm^2^ and (b) 3 nondiffusion weighted images (*b* = 0 s/mm^2^).

The original DICOM images were converted into the Nifti format using the Dcm2Nii (MRIcroN, version 4/2010) script. All subsequent pre‐processing steps were performed using the FMRIB Software Library (FSL, version 5.0, http://fsl.fmrib.ox.ac.uk/fsl/). Initial steps included brain‐tissue extraction and correction for possible eddy‐current induced distortions. A diffusion tensor model was applied to each voxel using DTIFIT to calculate maps for fractional anisotropy (FA); mean diffusivity (MD); axial diffusivity (AD, corresponding to the first eigenvector L1); and radial diffusivity (RD, calculated as average of the L2 and L3 maps). All FA images were visually inspected (by R. S.) and excluded if any image artifacts were observed.

Additional processing followed the standardized pipeline for TBSS analyses. First, each subject's FA image was nonlinearly registered to a standard FA template (http://fsl.fmrib.ox.ac.uk/fsl/fslwiki/FMRIB58_FA), and then averaged to a study‐specific template to which each subject's FA image was subsequently nonlinearly registered. Thereafter, a mean FA image was created, thresholded at 0.2 and thinned to obtain a mean FA skeleton. These projection‐steps upon the FA skeleton were subsequently also applied to the other non‐FA data images (MD, AD, and RD).

### 2.4 Statistical analyses

All statistical analyses were performed using MATLAB (version R2009b, http://ch.mathworks.com). The Lilliefors test was applied to test for normality distribution of demographic and clinical variables. The *t* test, Mann–Whitney *U* test, or chi‐square tests were used for between‐group comparisons where appropriate.

A voxel‐wise general linear model was applied to the skeletonized DTI images (FA, MD, AD, and RD), using permutation‐based nonparametric testing. Appropriate designs were applied for case–control (ALS vs. controls) and ALS subgroup comparisons and the demographic variables age and gender were as a minimum included in all designs as possibly confounding covariates. For subgroup comparisons, patients were first stratified based on their rD50‐derived disease Phase (Phase I vs. Phase II; additionally corrected for D50 as covariate) and second for their total disease aggressiveness (high = D50 < 30 months vs. low = D50 ≥ 30 months; additionally corrected for cFS and onset‐type as covariates). For reasoning of the chosen covariates for the subgroup analyses readers are referred to Supplementary Table S2.

Additional voxel‐wise regression analyses were performed using symptom duration and parameters of the D50 model for the entire ALS cohort. To further clarify the results of the analyses mentioned above, subsequent regression analyses have been conducted with the symptom duration, D50, cFS, and cFL within the subgroups of Phase I and Phase II patients, respectively.

For comparison purposes, we conducted analyses within the subcohort of patients who had received ALSFRS‐R assessment nearby the day of MRI acquisition (*n* = 114). Here, regression analyses with the parameters cFS and D50 were calculated and compared to regressions with the original ALSFRS‐R and the PR (calculated as: [48‐ALSFRS‐R]/symptom duration).

The threshold‐free cluster enhancement method (Winkler, Ridgway, Webster, Smith, & Nichols, 2014) was applied and *p*‐values are reported as family‐wise error‐corrected to account for multiple comparisons. Statistical significance was set at *p* < .001 for case–control comparisons and voxel‐wise regressions within the ALS‐cohort and at *p* < .05 for all ALS subgroup analyses.

## 3 RESULTS

### 3.1 Study cohort

Detailed clinical and demographic data for patients with ALS (*n* = 145) are outlined in Table 1. While controls were younger than patients (ALS: median 65 years, interquartile range 14.25; controls: median 53.7 years, interquartile range 13.4; *p* < .001), no significant differences in gender distribution (controls: 35 females, 50.7%; *p* = .419) or distribution of handedness (controls: 5 left‐handed, 7.2%; *p* = .948) were noted.

**TABLE 1:** Demographic and clinical data for patients with ALS

Characteristics	ALS, *n* = 145		
*Demographic*			
Age at MRI (in years) ‡	65 ± 14.2 (31.83–81.41)		
Gender (male/female) §	80/65, 55.2%/44.8%		
Handedness (left/right/unknown) §	12/126/7, 8.3%/86.9%/4.8%		
*Core clinical data*			
Riluzole intake at MRI (yes/no) §	114/31, 78.6%/21.4%		
Relevant Riluzole intake in the disease course (>50%) (yes/no) §	108/37, 74.5%/25.5%		
Symptom duration at MRI (in months) ‡	13 ± 12.5 (2–65)		
Onset‐type (limb/bulbar) §	100/45, 69%/31%		
*D50 disease progression model parameters*			
D50 (months) ‡	28.8 ± 20.6 (3.51–86.4)		
dx ‡	11.33 ± 8.87 (1.37–35.48)		
rD50 at MRI †	0.28 ± 0.13 (0.05–0.7)		
Phase I at MRI (0 ≤ rD50 < 0.25) §	I	56	38.6%
Phase II at MRI (0.25 ≤ rD50 < 0.5) §	II	85	58.6%
Phase III/IV at MRI (rD50 ≥ 0.5) §	III/IV	4	2.8%
cFS at MRI (points) †	37.81 ± 6.26 (15.13–48.53)		
cFL at MRI (points lost per month) ‡	0.81 ± 0.74 (0.12–7.07)		

*Note:* Continuous data are summarized for † as mean ± *SD* or for ‡ as median ± interquartile range (each with the total range in brackets). For § categorial data, the number of cases and percentages are given. Some variables are time‐point dependent throughout patients' individual disease course and refer to the day of MRI acquisition (as labeled with “at MRI”), the others depict constant characteristics of patients' overall disease course. Riluzole intake is reported in the context of (a) at MRI acquisition (b) a relevant intake (>50%) of 100 mg/day throughout the entire disease course (from symptom‐onset until end of study/time of death).

Abbreviations: ALS, amyotrophic lateral sclerosis; cFL, calculated functional loss; cFS, calculated functional state; D50, estimated time in months for an individual to lose 50% of functionality; MRI, magnetic resonance imaging; rD50, relative D50.

Within the ALS cohort, 56, 85, and 4 patients were in disease Phases I, II, and III, respectively. Patients in Phase I had lower disease aggressiveness (higher D50) and were on average younger and more frequently male relative to patients in Phase II. Patients were also stratified based on their overall disease aggressiveness (individual D50 values). Patients with high aggressiveness (D50 < 30 months) were older, had a lower cFS and presented more frequently with bulbar onset than those with low aggressiveness (D50 > 30 months).

All of the above‐mentioned potential confounding variables were subsequently included as nuisance covariates for the intergroup TBSS analyses. For an overview of the subgroups and reasoning for the chosen covariates, see Supplementary Table S2.

### 3.2 Widespread WM changes in ALS patients relative to controls

**FIGURE 2:**
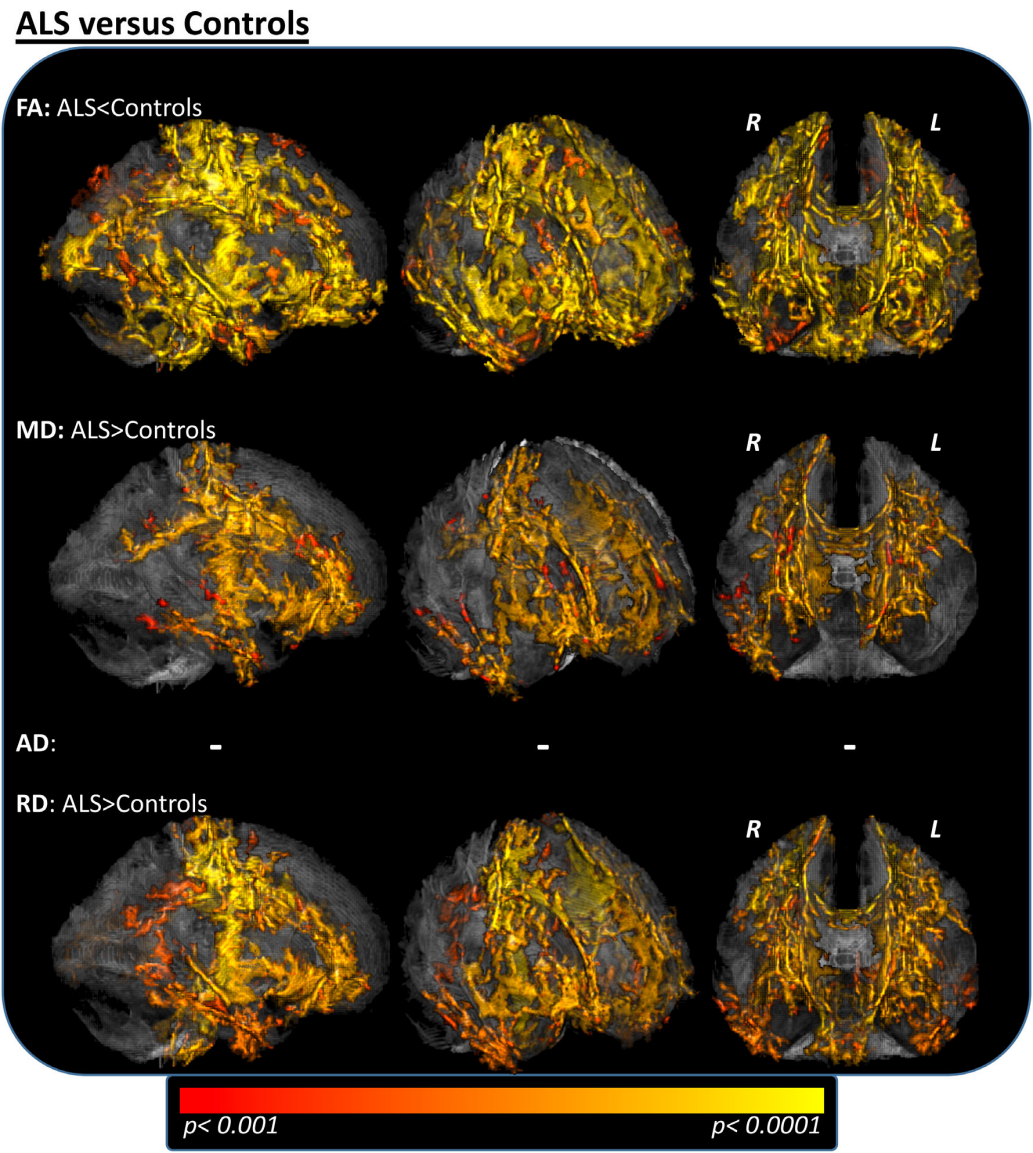
Between‐group differences for patients and healthy controls. Tract‐based spatial statistics (TBSS) between‐group comparisons of patients (*n* = 145) and healthy controls (*n* = 69) revealed widespread ALS‐related WM changes. These were most evident in decreased FA values, mainly located at bilateral CSTs, body/genu of the corpus callosum, adjacent corona radiata, and WM of bilateral frontal, parietal, temporal, occipital lobes, brainstem, and right‐cerebellar pathways. RD increases for the ALS cohort were colocalized with the FA changes (although less widespread), while the MD increases affected mainly the bilateral CSTs and the body of the corpus callosum. When applying the same significant threshold, no differences were noted for AD between patients and controls. TFCE; FWE corrected *p* < .001; nuisance covariates: age and gender. AD, axial diffusivity; ALS, amyotrophic lateral sclerosis; CST, corticospinal tract; FA, fractional anisotropy; FWE, family‐wise error; L, left hemisphere; MD, mean diffusivity; R, right hemisphere; RD, radial diffusivity; TFCE, threshold‐free cluster enhancement; WM, white matter

At the case–control level, patients with ALS exhibited widespread decreases of FA in several WM tracts (Figure 2; *p* < .001; corrected for age and gender), including the bilateral CST, body/genu of the corpus callosum and adjacent corona radiata as well as brainstem and cerebellar pathways. Additional FA decreases were observed in bihemispheric frontal, parietal, temporal, and occipital lobes; these included long association tracts like the bilateral superior/inferior longitudinal, uncinate, and fronto‐occipital fasciculi. In patients, regions with significant FA decreases also displayed increased RD relative to controls. MD increases within the case–control contrast were mainly restricted to bilateral CSTs, body, and splenium of the corpus callosum with adjacent corona radiata and the right inferior longitudinal fasciculus.

No significant between‐group differences for AD contrasts, FA increases, or MD/RD decreases were noted.

### 3.3 Subgroup comparisons between disease Phases I and II and between low and high disease aggressiveness

No significant differences for any contrast (FA, MD, AD, or RD) were observed for the subgroup comparisons between patients in disease Phases I and II (*p* < .05, corrected for age, gender and D50); these results remained unchanged by the inclusion of patients from Phase III (*n* = 4).

**FIGURE 3:**
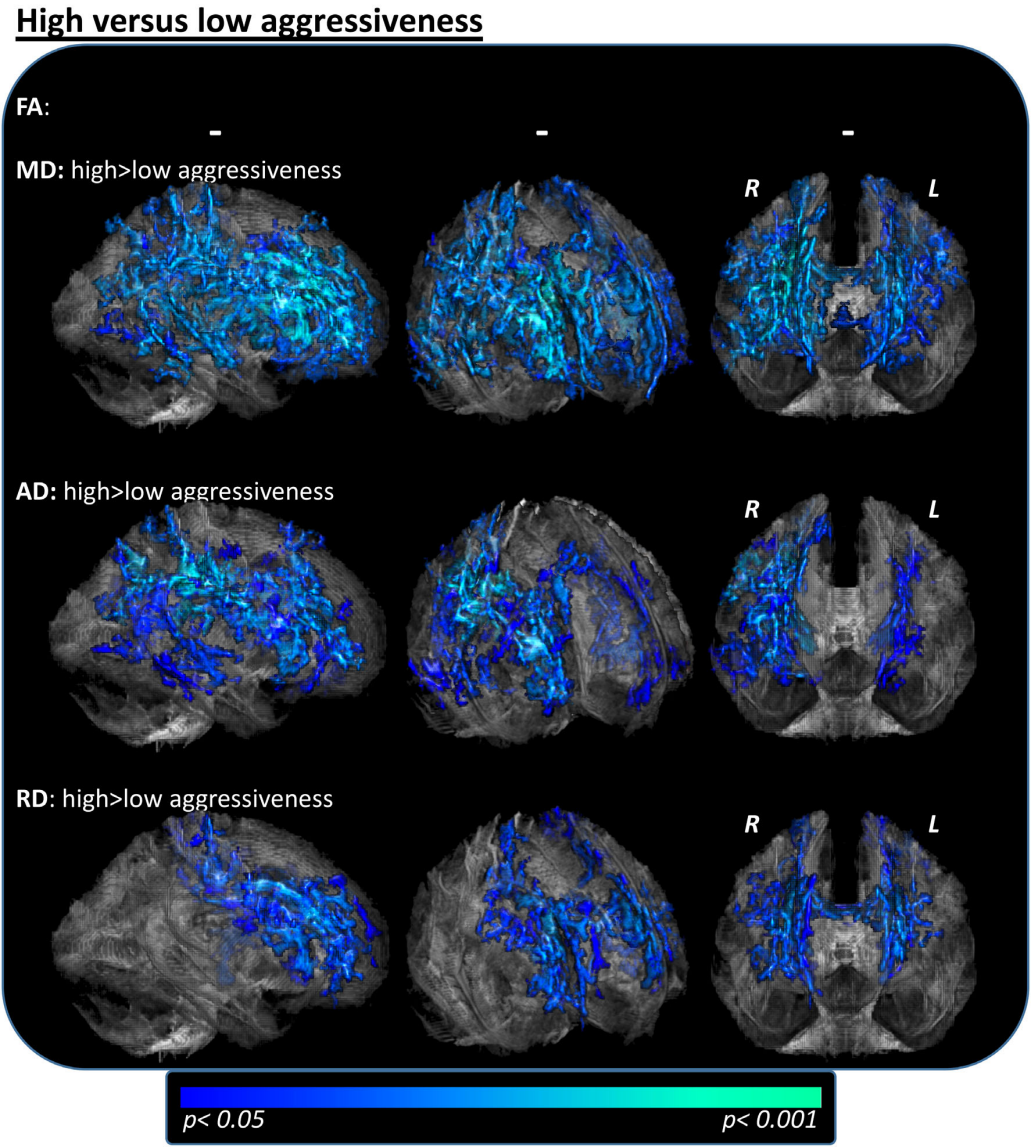
Tract‐based spatial statistics (TBSS) subgroup analyses of patients with high aggressiveness (D50 < 30 months, *n* = 79) versus low aggressive ALS (D50 ≥ 30 months, *n* = 66). High aggressiveness was associated with increases in MD and AD mainly in frontal and parietal long association tracts and emphasized in the right hemisphere. The RD increases were borderline significant and mainly localized at the body of the corpus callosum and upper CSTs. TFCE; FWE corrected *p* < .05; nuisance covariates: age, gender, onset‐type, cFS. AD, axial diffusivity; ALS, amyotrophic lateral sclerosis; ALSFRS‐R, ALS Functional Rating Scale (Revised); CST, corticospinal tract; D50 estimated time in months for an individual to lose 50% of functionality; FA, fractional anisotropy; FWE, family‐wise error; L, left hemisphere; MD, mean diffusivity; R, right hemisphere; RD, radial diffusivity; TFCE, threshold‐free cluster enhancement; WM, white matter

Subgroup comparisons based on disease aggressiveness revealed elevated DTI values in patients with high aggressiveness (D50 < 30 months) (Figure 3; *p* < .05; corrected for age, gender, onset‐type, and cFS). MD changes were primarily localized in the bifrontal, biparietal, and right‐temporal lobes and both CSTs, while elevated AD values were mostly restricted to deep frontal/parietal WM and were particularly pronounced in the right hemisphere. RD contrast clusters were primarily observed within the callosal body and frontal WM. No significant differences were observed for FA values.

### 3.4 Voxel‐wise regression analyses with clinical parameters

**FIGURE 4:**
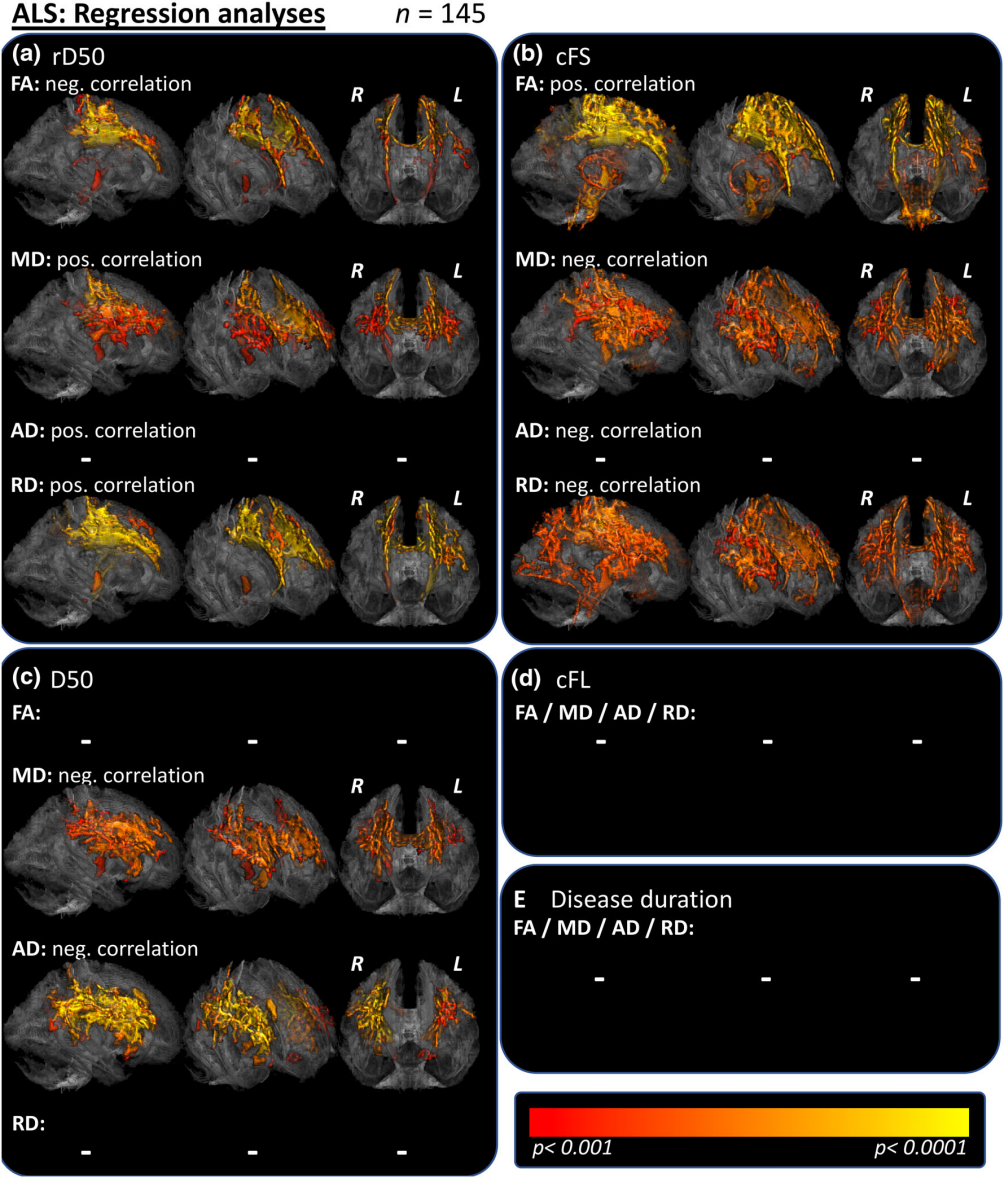
Voxel‐wise regression analyses with clinical parameters. (a) Voxel‐wise regression revealed a negative correlation of rD50 with FA and basically colocalized positive correlation with RD/MD, mainly for the body of the corpus callosum and adjacent corona radiate as well as upper parts of the CSTs. No correlations with AD could be seen for this significance level. (b) Voxel‐wise regressions with the parameter cFS were more extensive but in principle colocalized to the ones with the parameter rD50. (c) Regression analyses with the parameter D50 revealed negative correlations that were most significant for AD, followed by MD. These were mainly located at bilateral long association tracts of the frontal and parietal lobes. (d/e) Regression analyses with the cFL or the symptom duration did not reveal any significant correlations. TFCE; FWE corrected *p* < .001. Dash signs (−) mark missing correlations. AD, axial diffusivity; ALS, amyotrophic lateral sclerosis; CST, corticospinal tract; cFL, calculated functional loss; cFS, calculated functional state, each at the time of MRI; D50, estimated time in months for an individual to lose 50% of functionality; FA, fractional anisotropy; FWE, family‐wise error; L, left hemisphere; MD, mean diffusivity; rD50, relative D50 at the time of MRI; R, right hemisphere; RD, radial diffusivity; TFCE, threshold‐free cluster enhancement; WM, white matter

Regression analyses between clinical parameters and DTI measures were conducted for the entire ALS cohort (Figure 4; *p* < .001), and for the subgroups of patients in Phase I (Table 2; Supplementary Figure S2; *p* < .05) and Phase II (Table 2; Supplementary Figure S3; *p* < .05).

**TABLE 2:** Voxel‐wise regression analyses within disease phases

ALS patients in Phase I, *n* = 56		
rD50	FA	—
	MD (pos.)	GCC(B), BCC(B), SCC(B), ALIC(B), PLIC(B), RLIC(L), ACR(B), PCR(B), SCR(B), PTR(B), EC(B), CG(L), SLF(B), SFOF(B)
	AD (pos.)*	GCC(B), BCC(B), SCC(B), ALIC(B), PLIC(B), RLIC(B), ACR(B), PCR(B), SCR(B), PTR(B), EC(B), CG(B), Fornix(B), SLF(B), SFOF(B)
	RD (pos.)	GCC(B), BCC(B), SCC(B), ALIC(L), PLIC(L), ACR(B), PCR(B), SCR(B), EC(L), CG(B), SFOF(L)
cFS	FA (pos.)	GCC(L), BCC(L), ACR(L), SCR(L)
	MD (neg.)*	Widespread correlations including bihemispheric fibertracts of all cerebellar lobes, brainstem, and cerebellum (assignable to all ICBM‐DTI‐81 white‐matter labels)
	AD (neg.)*	
	RD (neg.)	
D50	FA/MD/RD	—
	AD (neg.)*	ALIC(R), PLIC(R), RLIC(R), ACR(R), PCR(R), SCR(R), PTR(R), EC(R), SLF(R), SFOF(R)
cFL	FA (neg.)	GCC(B), BCC(B), SCC(B), ACR(B), PCR(R), SCR(B), CG (L)
	MD (pos.)*	GCC(B), BCC(B), SCC(B), ACR(B), PCR(B), SCR(B), ALIC(B), PLIC(B), RLIC(R), PTR(R), EC(B), CG(B), SLF(B), SFOF(B)
	AD (pos.)*	
	RD (pos.)	GCC(B), BCC(B), SCC(B), ACR(B), PCR(R), SCR(B), EC(R), CG(L), SLF(R)
SD	FA/MD/AD/RD	—
ALS patients in Phase II, *n* = 85		
rD50	FA/MD/RD	—
	AD (pos.)*	MCP(B)
cFS	FA/RD	—
	MD/AD (neg.)*	MCP(B), ICP(R)
D50	FA/MD/RD	—
	AD (neg.)*	BCC(B), ACR(B), PCR(B), SCR(B), ALIC(B), PLIC(B), RLIC(B), PTR(L), EC(B), SLF(B), SFOF(B)
cFL	FA/MD/AD/RD	—
SD	FA/MD/AD/RD	—

*Note:* Fibertracts have been identified based on the ICBM‐DTI‐81 white‐matter labels atlas in the FMRIB Software Library (FSL, v5.0). *Asterisks mark the contrasts that contained the most‐significant suprathreshold‐voxels for the regression analysis with the respective disease metric.

Abbreviations: ACR, anterior corona radiata; ALIC, anterior limb of internal capsule; B, bihemispheric; BCC, body of corpus callosum; CG, cingulate gyrus; GCC, genu of corpus callosum; ICP, inferior cerebellar peduncle; L, left hemisphere; MCP, middle cerebellar peduncle; PCR, posterior corona radiata; PLIC, posterior limb of internal capsule; PTR, posterior thalamic radiation; R, right hemisphere; RLIC, retrolenticular part of internal capsule; SCC, splenium of corpus callosum; SCR, superior corona radiata; SD, symptom duration; SFOF, superior fronto‐occipital fasciculus; SLF, superior longitudinal fasciculus.

Regression‐contrasts for the entire cohort noted significant correlations for rD50 in FA, MD and RD contrasts (Figure 4a), that presented in principle similar as the respective contrasts with the parameter cFS, although more widespread patterns in the affected fibertracts were revealed for the latter. The cFS correlated positively and negatively with FA and RD, respectively, in regions that were previously noted as relevant at the case–control level (compare to Figure 2), particularly in the bilateral CST and corpus callosum. A significant inverse correlation between the cFS and the MD was observed within the bihemispheric fronto/parietal and temporal pathways and thus also within the same regions that showed MD alterations at the case–control level (Figure 4b). Disease aggressiveness analyses revealed negative correlations between D50 and MD/AD in the bilateral frontal and parietal pathways for the entire ALS cohort (Figure 4c). This was in keeping with the results of the subgroup analyses using stratification by D50 (compare to Figure 3). No correlations between DTI parameters and the cFL or the symptom duration were noted for the entire ALS cohort (Figure 4d,e).

For the subgroup of patients in Phase I, again similar patterns of correlations were revealed comparing the contrasts of correlations with either the rD50 or the cFS (Table 2). Again, voxel‐wise regressions for the cFS showed the more widespread significant correlation patterns, mainly for the AD, MD, and RD. Regression analyses with the parameter D50 revealed solely a negative correlation with AD in right subfrontal WM. The cFL showed even more extensive correlations with AD/MD in bifrontal WM in Phase I patients; the RD (positive correlation with cFL) and FA (negative correlation with cFL) showed effects that were essentially restricted to the body of the corpus callosum. No correlations with the symptom duration were noted for patients in Phase I for any contrast. For patients in Phase II, negative correlations with the rD50 or cFS could only be shown restricted to the bilateral cerebellum, these were most evident within the vermis and crura I cerebelli. rD50 showed a positive correlation with AD and cFS conversely a negative correlation with AD as well as negative correlation in the MD contrast. The aforementioned correlations with the parameter D50 (for all ALS patients or Phase I patients) were again detectable for the Phase II patients, as bihemispheric subfrontal AD elevations that were associated with higher disease aggressiveness (i.e., lower D50, negative correlation). Regression analyses with the cFL or the symptom duration again revealed no significant results for the Phase II subcohort.

**FIGURE 5:**
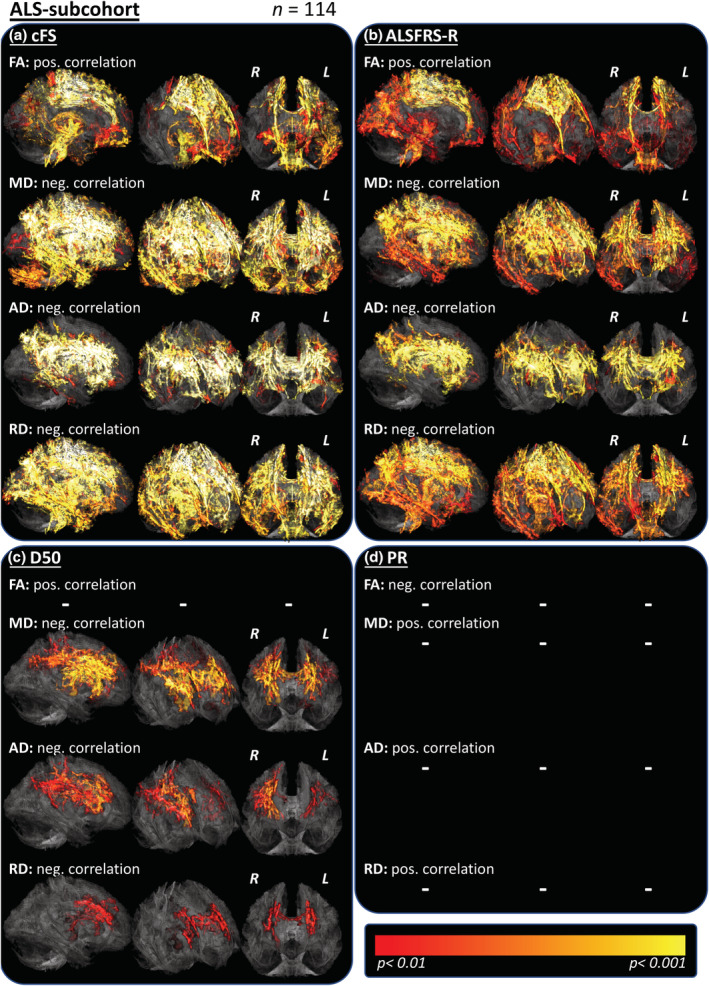
Comparison of D50 model parameters with the ALSFRS‐R and the PR. The regression analyses were conducted in the subcohort of patients with timely available ALSFRS‐R (assessed with 10 days prior to or after MRI; *n* = 114). (a,b) The cFS showed widespread correlations with DTI metrics (as before) that were localized in the same fibertracts as in the respective correlation contrasts with the ALSFRS‐R. (c) The D50 value revealed again negative correlations with MD more than AD more than RD. D) No significant correlations with the PR could be revealed for any DTI metric, which indicates the superiority of the D50 value to measure disease aggressiveness in this ALS cohort. TFCE; FWE corrected *p* < .01. Dash signs (−) mark missing correlations. AD, axial diffusivity; ALS, amyotrophic lateral sclerosis; cFS, calculated functional state (at the time of MRI); D50, estimated time in months for an individual to lose 50% of functionality; FA, fractional anisotropy; FWE, family‐wise error; L, left hemisphere; MD, mean diffusivity; MRI, magnetic resonance imaging; rD50, relative D50 (at the time of MRI); R, right hemisphere; RD, radial diffusivity; TFCE, threshold‐free cluster enhancement

For the subgroup of 114 ALS patients who received timely nearby ALSFRS‐R scoring (assessed within 10 days before or after the MRI; Supplementary Table S1), voxel‐wise regressions with this score revealed only a small cluster beyond the precentral gyrus of positive correlation with the FA if applying the strict significance level of *p* < .001 (data not shown). For illustration purposes, the significance threshold was lowered to *p* < .01, what revealed patterns of correlations with the ALSFRS‐R that anatomically were well colocalized with the contrasts of correlations with the cFS (Figure 5a,b). Again, significant and extensive patterns could be revealed in correlation contrasts with the D50 value, most important for MD and AD contrasts and also to a smaller extent for the RD (Figure 5c). For the PR, no significant correlations could be shown with any DTI‐measure for this group of 114 ALS patients (Figure 5d).

## 4 DISCUSSION

**FIGURE 6:**
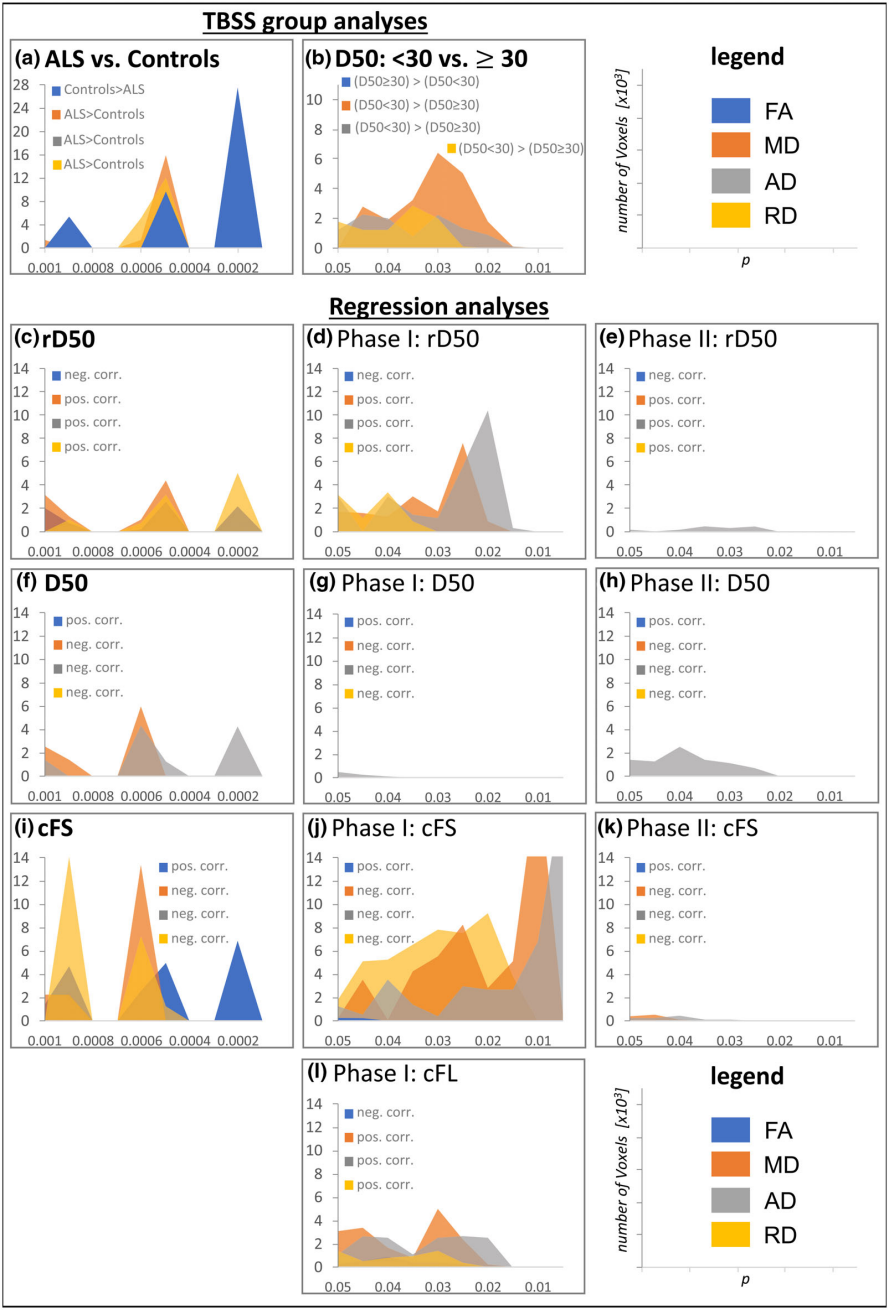
Summary of significant tract‐based spatial statistics (TBSS) results in association with the D50 model. The plots refer to suprathreshold Voxels of significant results for the respective TBSS analyses. The histograms show the number of voxels for the respective *p*‐value level (with increasing significance from left to right on the x‐axis). (a,c–e) Comparing ALS patients to healthy controls revealed the most relevant significant differences for FA (a). However, accumulated disease (rD50) did not correlate with the FA, but with MD, RD and AD (c), for the latter being most evident for the subcohort of Phase I patients (d) and still detectable within Phase II patients (e). No significant differences were noted when comparing patients in Phase I with those in Phase II (data not shown). (b,f–h) The subcohort comparison of patients' D50‐derived overall disease aggressiveness revealed the most significant differences for MD and AD (b), which was comparable in regression analyses within the entire ALS cohort (f). For AD, this was mainly driven by the Phase II patients (h), while only minor AD correlations were noted for Phase I patients (g). Again, no associations between FA and D50 were observed. In general, the results showed stable associations between increased AD and higher disease aggressiveness (lower D50), located in long fronto‐central associations tracts. (i–k) The regression analyses with the parameter cFS showed in principle comparative patterns as in the concerning analyses with the parameter rD50, but in addition also relevant correlations with FA (i). These were additionally confirmed within the subgroup of Phase I patients (j) and for Phase II patients there were again only minor correlations with AD/MD (in bilateral cerebellum). (l) Significant correlations with the parameter cFL were found only within the subcohort of Phase I patients, not for Phase II patients or the entire ALS‐cohort, which again underlines the capability of DTI to detect changes even during the relatively early stable disease Phase I. ALS, amyotrophic lateral sclerosis; D50, estimated time in months for an individual to lose 50% of functionality; cFL, calculated functional loss, cFS, calculated functional state, rD50, relative D50, each at the time of MRI

The present study is the first to comprehensively evaluate tract‐related structural integrity in patients with ALS by distinguishing between individual accumulated disease/disease covered and disease aggressiveness. These disease characteristics were defined and assessed using the novel D50 model. Figure 6 gives a pictorial overview of the significant results that have been revealed, in particular comparing the significance levels of the different DTI metrics (FA, MD, AD, and RD) for the respective contrasts.

DTI metrics differed significantly between patients with ALS and healthy controls, in both motor and extra‐motor pathways, indicating widespread WM pathology in ALS. Decreased FA and increased RD were the most pronounced ALS‐associated changes; colocalization of the affected WM‐tracts was evident in both contrasts. This is in line with prior TBSS studies with moderate‐to‐big sample sizes that also reported widespread differences—particularly in RD and FA contrasts—in case–control comparisons (Bao et al., 2018; Ben Bashat et al., 2011; Cardenas‐Blanco et al., 2014; Cirillo et al., 2012; de Albuquerque et al., 2017; Sarica et al., 2014; Zimmerman‐Moreno et al., 2016). However, some studies using smaller cohorts have reported that AD was the most profoundly altered DTI metric within their patient groups (Geraldo et al., 2018; Metwalli et al., 2010).

Altogether, the results of the current study show RD/FA changes within the core‐tracts of ALS WM pathology that corroborate the findings of a recent meta‐analysis of 14 DTI studies by Zhang et al. (2018). There, decreases of FA were reported in the corpus callosum, the corona radiata, the CSTs, and superior longitudinal fasciculi. Overall, FA reductions in the CST are consistently and frequently reported in TBSS case–control analyses (Alshikho et al., 2016; Cardenas‐Blanco et al., 2016; Trojsi et al., 2019; Zhang et al., 2018).

### 4.1 DTI changes are associated with disease accumulation in early disease Phase I

The D50 model enabled a pseudo‐longitudinal comparison of patients in early stable vs. early progressive rD50‐derived disease Phases. Using rD50, the effect of accumulated disease—independent of individual disease aggressiveness—is assessed separately. While no significant differences in DTI metrics were noted between patients in Phases I and II, significant correlations with both rD50 and cFS, especially for the FA and RD contrasts, were observed. Comparably, previous TBSS studies (with moderate cohort sizes; *n* = 19–54; Supplementary Table S3) that performed voxel‐wise regression analyses primarily reported correlations between FA values and ALSFRS‐R scores (Cirillo et al., 2012; de Albuquerque et al., 2017; Prudlo et al., 2012; Sage et al., 2009; Trojsi et al., 2015). Conversely, studies with smaller cohorts (*n* = 12–15) reported no significant correlations with the ALSFRS‐R (Geraldo et al., 2018; Metwalli et al., 2010; Rose et al., 2012), which likely demonstrates a sample size effect. Given that our regression analyses revealed no additional changes in the supratentorial tracts (no changes for FA/RD noted either) for patients in Phase II, these results suggest that disease accumulation‐driven changes primarily occur in Phase I (Table 2; Supplementary Figure S3a,b). In keeping with this, direct subgroup comparisons between Phase I and II patients did not reveal any difference in DTI metrics. This may potentially explain why Keller et al. (2011) reported no correlations between FA values and ALSFRS‐R scores for their cohort of 33 patients; it is possible that only patients in Phase II were analyzed given that the highest reported ALSFRS‐R score was 40 points (compare to Figure 1c). Here we show that in Phase II, the only significant correlations noted are within bilateral cerebellum between the cFS and MD/AD elevations and rD50 and AD increases. Cerebellar involvement in ALS has been previously reported by DTI studies (Keil et al., 2012; Menke et al., 2018; Prell et al., 2013; Trojsi et al., 2015); our data indicate that the dynamic structural changes in the cerebellum are a feature of ongoing disease in Phase II.

Of particular interest is the behavior of measurable FA changes across the disease course: although significant and widespread FA decreases reflect ALS‐driven pathology at the case–control level (Figures 2 and 6a), no further FA changes were noted (a) between Phases I and II and (b) within regression analysis with the rD50 (that displays individual disease covered, normalized for a patient's disease aggressiveness = D50; Figure 6c–e), or in regression analyses with any parameter for the subcohort of Phase II patients (Table 2; Supplementary Figure S3 and Figure 6e/h/k). In summary, when applying whole‐brain analysis of skeletonized WM, we noted no evidence to suggest that additional FA alterations are detectable in disease Phase II. This may reflect a “saturation” effect of the FA parameter for the majority of neuronal fibertracts wherein significant neurodegenerative processes have already occurred at earlier (possibly presymptomatic) disease stages.

We demonstrated that the D50 model is well suited for cross‐sectional analyses and allows comparisons between highly heterogeneous patients. One of the advantages of this approach is the ability to include patients who did not receive ALSFRS‐R assessment at the time of MRI acquisition. Traditionally, these patients would have been excluded from further analyses, thus bearing the potential of selection bias. We here used the cFS instead, which showed stable associations with DTI‐signals (Figures 4 and 5b). We further show, that the correlations revealed with the cFS are comparable to those with the original ALSFRS‐R scores in the respective subgroup analyses (Figure 5a,b). The reliability of the parameter cFS is further supported by the observation that the averaged differences between model‐derived cFS and original ALSFRS‐R scores per patient had a median of −0.1 points (interquartile range 0.25; total range: −1.68 to 0.72; Figure 1d). We conclude that the cFS is a usable marker for a patient's remaining functionality, which can be calculated for any given time point and decreases the noise typically associated with ALSFRS‐R assessments, for example, due to different raters (Bakker et al., 2020; Franchignoni et al., 2013).

Our approach may also help guide future studies aimed at longitudinally analyzing TBSS correlates of ALS‐related functional loss. These are more promising if patients receive their MRIs during Phase I; this may have been the case in the study conducted by de Albuquerque et al. (2017), who reported a progressive increase of MD and AD in the corpus callosum after 8 months. Similarly, Stampfli et al. (2019) noted progressive FA decreases in several right‐hemispheric fibertracts after 3–6 months. Conversely, Alruwaili et al. (2019) observed no FA/MD differences within a cohort of 16 ALS patients after 6 months; this may have been due to the minimal decline in functionality over the study duration (mean ALSFRS‐R dropped from 39 to 38 points). A second issue for longitudinal studies arises from the application of fixed time‐intervals for patients with vastly differing progression types, which can often occlude biological signals, given that functionality declines across variable timeframes. We therefore posit, as have previous studies, that absolute symptom duration is not an appropriate disease metric in an ALS cohort as no significant associations with it have been noted (de Albuquerque et al., 2017; Geraldo et al., 2018; Metwalli et al., 2010; Trojsi et al., 2015).

### 4.2 Stable signatures of ALS disease aggressiveness in WM tracts revealed with DTI

We further demonstrated that individuals with higher overall disease aggressiveness (i.e., lower D50) display DTI changes within established core regions of ALS pathology. We observed substantial MD and AD elevations in fronto–parietal long association tracts. The involvement of these pathways has been previously reported; AD elevations are often concomitant with cognitive and/or behavioral impairment (Alruwaili et al., 2018; Kasper et al., 2014), both of which have been noted in patients with more rapidly progressive disease forms (Bock et al., 2017; Calvo et al., 2017).

In the D50 model, overall disease aggressiveness is captured by parameter D50 whereas disease activity at the time of MRI is represented by cFL; cFL yielded significant correlations for patients in Phase I and not those in Phase II or the entire ALS cohort. This is curious, regarding the relatively low interindividual range of the parameter cFL in Phase I, while cFL variance substantially increases for Phase II patients (Supplementary Table S2). Again, this underscores DTI's sensitivity toward even subtle changes during very early disease.

Here, using cFL (indicator of local disease activity) and D50, we have demonstrated consistent and robust associations between DTI‐quantified changes and disease aggressiveness in ALS. Similar analyses have been previously performed using the PR parameter that has inherent limitations. As an index, the PR presumes that progression in ALS is linear and thus does not fully capture the patients' progression throughout the whole disease course (Franchignoni et al., 2013; Gordon et al., 2010; Simon et al., 2014). This may explain why in the regression analyses of patients with an available ALSFRS‐R no significant correlations with the parameter PR could be revealed, while D50 showed stable correlations (Figure 5c,d). Unsurprisingly, the majority of prior TBSS studies did also not observe any relevant voxel‐wise correlations between the PR and DTI metrics (Alshikho et al., 2016; Geraldo et al., 2018; Kopitzki et al., 2016; Rose et al., 2012; Senda et al., 2017). Cirillo et al. (2012) noted an inverse correlation between FA and the PR within the CSTs (pons) and frontotemporal pathways, and a positive correlation for both the RD and MD (e.g., in bilateral inferior longitudinal and uncinate fasciculi; *p* < .001, uncorrected). Other studies utilized an acute or “local” PR; however, neither Senda et al. (2017)) who assessed 6‐month‐follow‐ups, nor de Albuquerque et al., 2017) who assessed changes over an 8‐month period reported any correlations with the local PR in their TBSS studies.

Here, we provide a rational foundation for the use of D50 model‐derived parameters, especially since the D50 value itself captures patients' disease aggressiveness based on their individual disease trajectories.

### 4.3 Limitations

The present study is not without its limitations. Standardized neuropsychological and genetic profiles were unavailable for the entire cohort. Nevertheless, we posit that given its size, the cohort is representative of the variance within the regional patient population. Accordingly, the distribution of representative disease characteristics mirrored all the ALS patient data available at our neuromuscular center (Figure 1d).

## 5 CONCLUSION

The present study has demonstrated the potential of the D50 model for characterizing highly heterogenous cross‐sectional ALS cohorts and generating informative pseudo‐longitudinal data while accounting for strongly varying phenotypic presentation. Within this framework, we show that TBSS analyses are particularly sensitive toward disease accumulation‐driven changes in early disease (Phase I). Second, characteristic structural WM changes are strongly associated with higher disease aggressiveness. Taken together, these observations suggest that DTI reflects disease activity, which appears to remain stable over time. Therefore, DTI may constitute a robust readout for the detection of treatment‐induced reduction of disease activity prior to obvious clinical improvement.

Finally, we show that longitudinal clinical data capture in ALS should be tailored to individual disease trajectories, especially within mixed patient groups of differing progression types. We recommend using the D50 model to stratify patients in further studies developing or validating neuroimaging or other biomarkers for ALS.

## CONFLICT OF INTEREST

The authors declare no conflict of interest.

## Supporting information


**FIGURE S1** Consort diagram of participants
**FIGURE S2:** Voxel‐wise regression analyses within disease Phase I
**FIGURE S3**: Voxel‐wise regression analyses within disease Phase II
**TABLE S1:** Demographic and Clinical Data for patients with available
**TABLE S2:** Demographic and Clinical Data for patients in different sub‐groups.
**TABLE S3:** Previous TBSS studies with voxel‐wise regression analyses using the ALSFRS‐R

## Data Availability

The data that support the findings of this study are available from the senior author, upon reasonable request for scientific purpose.
