# Supplementary material for: Disease aggressiveness signatures of amyotrophic lateral sclerosis in white matter tracts revealed by the D50 disease progression model
Source: Hum Brain Mapp. 2020 Oct 26;42(3):737–52. doi: 10.1002/hbm.25258 (PMC7814763; doi:10.1002/hbm.25258)
Supplement: Supplementary file 1 — FIGURE S1 Consort diagram of participants FIGURE S2: Voxel‐wise regression analyses within disease Phase I FIGURE S3: Voxel‐wise regression analyses within disease Phase II TABLE S1: Demographic and Clinical Data for patients with available TABLE S2: Demographic and Clinical Data for patients in different sub‐groups. TABLE S3: Previous TBSS studies with voxel‐wise regression analyses using the ALSFRS‐R [file HBM-42-737-s001.docx]

**Supplementary material**

**Figure S1:** Consort diagram of participants

|  |
| --- |

**Figure S2:** Voxel-wise regression analyses within disease Phase I

A) Within the group of Phase I patients, widespread correlations with the parameter rD50 were revealed (most significantly for AD and MD)

B) Widespread correlations have been found with the parameter cFS, again most significant for AD and MD contrasts, whilst only minor effects for FA have been revealed within the left corona radiata.

C) AD showed a negative correlation with the D50 value in right-hemispheric fronto-parietal association-tracts.

D) The cFL showed extensive correlations within the group of Phase I patients, most emphasized as positive correlations with AD/MD in bifrontal WM.

Regression analysis with the symptom duration did not reveal any significant correlations (data not shown)

TFCE; FWE corrected *p* < 0.05. Dash signs (–) mark missing correlations.

*Abbreviations:* *ALS =* Amyotrophic Lateral Sclerosis; *AD =* Axial Diffusivity; *CST* = Cortico-Spinal-Tract; c*FL* calculated functional loss, c*FS* calculated functional state, each at the time of MRI; *D50* estimated time in months for an individual to lose 50% of functionality; *FA =* Fractional Anisotropy; *FWE =* Family-Wise Error; *L =* Left hemisphere; *MD =* Mean Diffusivity; *rD50* relative D50 at the time of MRI; *R =* Right hemisphere; *RD =* Radial Diffusivity; *TFCE =* Threshold-Free Cluster Enhancement*; WM =* White Matter.

**Figure S3**: Voxel-wise regression analyses within disease Phase II

A) In the sub-cohort of Phase II patients, a positive correlation of AD with the rD50 was found in cerebellar WM.

B) Negative correlations with the cFS were found only for AD/MD contrasts, again restricted to bilateral cerebellum.

C) AD showed a negative correlation with the D50 value in bi-hemispheric fronto-parietal association-tracts (more evident as for the Phase I patients, Fig. 5C).

D) No significant correlations with the cFL could be revealed for Phase II patients, as well as for the symptom duration (data not shown).

TFCE; FWE corrected *p* < 0.05. Dash signs (–) mark missing correlations.

*Abbreviations:* *ALS =* Amyotrophic Lateral Sclerosis; *AD =* Axial Diffusivity; *CST* = Cortico-Spinal-Tract; c*FL* calculated functional loss, c*FS* calculated functional state, each at the time of MRI; *D50* estimated time in months for an individual to lose 50% of functionality; *FA =* Fractional Anisotropy; *FWE =* Family-Wise Error; *L =* Left hemisphere; *MD =* Mean Diffusivity; *rD50* relative D50 at the time of MRI; *R =* Right hemisphere; *RD =* Radial Diffusivity; *TFCE =* Threshold-Free Cluster Enhancement*; WM =* White Matter.

| **Table S1:** Demographic and Clinical Data for patients with available  ALSFRS-R scores around MRI assessment | | | |
| --- | --- | --- | --- |
| **Characteristics** | **ALS, *n* = 114** | | |
| ***Demographic*** | | | |
| **Age** at MRI [in years] *‡* | 65.33 ± 16.2 (31.83 – 81.41) | | |
| **Gender** [male/female] *§* | 65/49, 57.0%/43.0% | | |
| ***Core and traditional clinical data*** | | | |
| **Disease duration** at MRI [in months] *‡* | 13.00 ± 13.0 (2 – 61) | | |
| **Onset-type** [limb/bulbar] *§* | 78/36, 68.4%/31.6% | | |
| **ALSFRS-R** [points with a maximum of 48] *‡* | 39.00 ± 10.0 (15 – 47) | | |
| **PR** [points lost per month] *‡* | 0.58 ± 0.6 (0.07 – 3.28) | | |
| **King’s Stage** at MRI [n per stage I-V] *§* | I:  II:  III:  IVa:  IVb:  V: | 35,  37,  32,  2,  8,  0, | 30.7%  32.5%  28.1%  1.8%  7.0%  0% |
| **MiToS Stage** at MRI [n per stage 0-V] *§* | 0:  I:  II:  III/IV/V: | 85,  27,  2,  0, | 74.6%  23.7%  1.8%  0% |
| ***D50 disease progression model parameters*** | | | |
| **D50** [months] *‡* | 28.99 ± 21.1 (3.51 – 84.22) | | |
| **dx** *‡* | 11.38 ± 9.8 (1.37 – 34.98) | | |
| **rD50** at MRI *†* | 0.28 ± 0.1 (0.05 – 0.7) | | |
| **Phase I** at MRI (0 ≤ rD50 < 0.25) *§*  **Phase II** at MRI (0.25 ≤ rD50 < 0.5) *§*  **Phase III/IV** at MRI (rD50 ≥ 0.5) *§* | I:  II:  III/IV: | 45  65  4 | 39.5%  57.0%  3.5% |
| **cFS** at MRI [points] *†* | 37.63 ± 6.5 (15.13 – 48.53) | | |
| **cFL** at MRI [points lost per month] *‡* | 0.80 ± 0.8 (0.16 – 7.07) | | |
| Continuous data is summarized for *†* as mean ± standard deviation or for *‡* as median ± interquartile range (each with the total range in brackets). For *§* categorial data, the number of cases and percentages are given. Some variables are time-point dependent throughout patients’ individual disease course and refer to the day of MRI acquisition (as labeled with ‘at MRI’), the others depict constant characteristics of patients’ overall disease course. For comparison purpose, traditionally used staging systems are given, as calculated from the ALSFRS-R score near to MRI. The King’s Staging system allocates patients to Stages I (involvement of one clinical region) until IV (respiratory or nutritional failure), whilst the MiToS System describes Stages 0 (functional involvement) until IV (loss of independence in four domains), the Stages V depict patients’ death respectively (Chio, Hammond, Mora, Bonito, & Filippini, 2015; Roche et al., 2012).  *Abbreviations:* ALS = Amyotrophic Lateral Sclerosis; ALSFRS-R = revised ALS functional rating scale (assessed within 10 days prior to or after MRI acquisition); D50 = estimated time in months for an individual to lose 50% of functionality; PR = Progression Rate, calculated as (48 – current ALSFRS-R)/months since symptom onset; cFS = calculated functional state; cFL = calculated functional loss; MRI = Magnetic Resonance Imaging; rD50 = relative D50 | | | |

**Table S2:** Demographic and Clinical Data for patients in different sub-groups.

|  | Phase I  (0 ≤ rD50  < 0.25) | Phase II  (0.25 ≤ rD50  < 0.5) | *p* | high aggressiveness  (D50 < 30) | low aggressiveness  (D50 ≥ 30) | *p* |
| --- | --- | --- | --- | --- | --- | --- |
| **Age** at MRI [in years] *‡* | 58.71 ± 15.7 (31.8 - 79.4) | 67.25 ± 9.7 (34.3 - 81.4) | <0.001* | 66.0 ± 13.8  (32.75 - 81.41) | 63.45 ± 15.0  (31.83 - 79.41) | 0.044* |
| **Gender** [male/female] *§* | 37/19,  66.1%/33.9% | 41/44,  48.2%/51.8% | 0.037* | 41/38,  51.9%/48.1% | 39/27,  59.1%/40.9% | 0.386 |
| **Onset-type** [limb/bulbar] *§* | 39/17,  69.6%/30.4% | 58/27,  68.2%/31.8% | 0.86 | 47/32,  59.5%/40.5% | 53/13,  80.3%/19.7% | 0.007* |
| **Disease duration**  at MRI [in months] *‡* | 9.0 ± 6.0  (4 - 31) | 16.0 ± 14.0  (2 - 65) | <0.001* | 11.0 ± 7.0  (2 - 27) | 23.0 ± 19.0  (4 - 65) | <0.001* |
| **rD50** at MRI  *†* | 0.14 ± 0.1 (0.05 - 0.23) | 0.35 ± 0.1 (0.25 - 0.49) | <0.001* | 0.3 ± 0.12  (0.06 - 0.7) | 0.25 ± 0.14  (0.05 - 0.51) | 0.053 |
| **D50** [months]  *‡* | 32.84 ± 22.3 (14.1 - 86.4) | 23.33 ± 17.7 (3.5 - 82.3) | <0.001* | 19.9 ± 9.0 (3.51 - 29.88) | 40.99 ± 24.1 (30 - 86.4) | <0.001* |
| **cFS** at MRI [points]  *†* | 43.75±2.2 (39.4 - 48.5) | 34.62 ± 4.3 (26.2 - 42.2) | <0.001* | 36.81 ± 5.8  (15.13 - 46.45) | 39.01 ± 6.6  (24.93 - 48.53) | 0.035* |
| **cFL** at MRI [points lost per month] *‡* | 0.47 ± 0.4 (0.12 - 1.25) | 1.14 ± 0.8 (0.41 - 7.07) | <0.001* | 1.25 ± 0.8  (0.42 - 7.07) | 0.52 ± 0.4  (0.12 - 1.11) | <0.001* |
| *†* continuous metric data is summarized as mean ± standard deviation and *p*-values refer to inter-sub-group t-tests; *‡* non-parametric data is depicted as median ± interquartile range and *p*-values refer to Mann-Whitney–U-tests (each with the total range in brackets). *§* for categorial data, the number of cases and percentages are given and *p*-values of inter-sub-group chi-square-tests. Some variables are time-point dependent throughout patients’ individual disease course and refer to the day of MRI acquisition (as labeled with ‘at MRI’), the others depict constant characteristics of patients’ overall disease course.  *Abbreviations:* ALS = Amyotrophic Lateral Sclerosis; D50 = estimated time in months for an individual to lose 50% of functionality; cFS = calculated functional state; cFL = calculated functional loss; MRI = Magnetic Resonance Imaging; rD50 = relative D50. | | | | | | |

**Table S3:** Previous TBSS studies with voxel-wise regression analyses using the ALSFRS-R

| **Citation** | **number of ALS patients** | **MRI field strength,**  **manufacturer,**  **number of gradient directions** | **ALSFRS-R** | **Correlations noted with the ALSFRS-R** |
| --- | --- | --- | --- | --- |
| de Albuquerque et al. (2017) | 53 | 3T,  N/A,  32 gradients | median 34.5  range 12-45 | - **FA**(pos.) >**RD**(neg.) >**MD**(neg.) correlations in fronto-parietal pathways (mainly CC) for p < 0.05 (corrected for age and gender)  - **no** for **AD**  - but also AD/MD increases (mainly in CC) in a longitudinal subcohort analysis |
| Trojsi et al. (2015) | 54 | 3T,  GE,  32 gradients | mean 33.4±7.9  range 16-47 | - for **FA** (pos. corr.) in the midpart of CC, superior and inferior longitudinal and fronto-occipital fasciculi and within WM underneath primary motor and premotor cortices, inferior frontal and temporal gyri, supramarginal gyri, visual cortices and brainstem for *p* < 0.05  - **no** correlations with **RD/MD** reported for *p* < 0.05  - AD not tested |
| Sage et al. (2009) | 28 | 3T,  Philips,  16 gradients | mean 39.7±6.3  range 17-48 | - **FA** (pos. corr.) in bilateral cranial parts of the CST and especially the (pre)frontal lobe for *p* < 0.05  - **MD** (neg. corr.) in CST, the hippocampal formations, the parietal and temporal lobe, and the cerebellum for *p* < 0.05  - AD/RD not tested |
| Prudlo et al. (2012) | 22 | 1.5T,  Siemens,  30 gradients | median 34  range 4–44 | **-** for **FA** (pos. corr) in widespread WM, supratentoriel, including both CSTs for *p* < 0.05  - MD/AD/RD not tested |
| Cirillo et al. (2012) | 19 | 3T,  GE,  32 gradients | mean 34.2±9.1  range 18-47 | - for **FA** (pos. corr.) in the WM underneath the left premotor cortex: the left paracentral lobule, the anterior cingulate and superior longitudinal fasciculus for *p* <0.05  - **no** correlations with **MD/AD/RD** reported for *p* < 0.05 |
| Keller et al. (2011) | 33 | 1.5T,  Siemens,  30 gradients | mean 34.5±3.7  range 24-40 | - **no** correlations for **FA** for *p* < 0.05 in a restricted volume of interest of upper brain parts  - MD/AD/RD not tested |
| Geraldo et al. (2018) | 14 | 3T,  Philips,  32 gradients | mean 42.86±2.7  range 38–46 | - **no** correlations for **FA/MD/AD/RD** found for *p* < 0.05 |
| Rose et al. (2012) | 15 | 3T,  Siemens,  60 gradients | mean 39±5.6  range 29–46 | - **no** correlations for **FA** found for *p* < 0.05  - MD/AD/RD not tested |
| Metwalli et al. (2010) | 12 | 3T,  Siemens,  64 gradients | 41.3±5.5  range 32-48 | - **no** significant correlations found for **FA/MD/AD/RD**  for p < 0.05 |

**References**

Chio, A., Hammond, E. R., Mora, G., Bonito, V., & Filippini, G. (2015). Development and evaluation of a clinical staging system for amyotrophic lateral sclerosis. *J Neurol Neurosurg Psychiatry, 86*(1), 38-44. doi:10.1136/jnnp-2013-306589

Cirillo, M., Esposito, F., Tedeschi, G., Caiazzo, G., Sagnelli, A., Piccirillo, G., . . . Trojsi, F. (2012). Widespread microstructural white matter involvement in amyotrophic lateral sclerosis: a whole-brain DTI study. *AJNR Am J Neuroradiol, 33*(6), 1102-1108. doi:10.3174/ajnr.A2918

de Albuquerque, M., Branco, L. M., Rezende, T. J., de Andrade, H. M., Nucci, A., & Franca, M. C., Jr. (2017). Longitudinal evaluation of cerebral and spinal cord damage in Amyotrophic Lateral Sclerosis. *Neuroimage Clin, 14*, 269-276. doi:10.1016/j.nicl.2017.01.024

Geraldo, A. F., Pereira, J., Nunes, P., Reimao, S., Sousa, R., Castelo-Branco, M., . . . de Carvalho, M. (2018). Beyond fractional anisotropy in amyotrophic lateral sclerosis: the value of mean, axial, and radial diffusivity and its correlation with electrophysiological conductivity changes. *Neuroradiology, 60*(5), 505-515. doi:10.1007/s00234-018-2012-6

Keller, J., Vymazal, J., Ridzon, P., Rusina, R., Kulist'ak, P., Malikova, H., . . . Jech, R. (2011). Quantitative brain MR imaging in amyotrophic lateral sclerosis. *MAGMA, 24*(2), 67-76. doi:10.1007/s10334-010-0237-4

Metwalli, N. S., Benatar, M., Nair, G., Usher, S., Hu, X., & Carew, J. D. (2010). Utility of axial and radial diffusivity from diffusion tensor MRI as markers of neurodegeneration in amyotrophic lateral sclerosis. *Brain Res, 1348*, 156-164. doi:10.1016/j.brainres.2010.05.067

Prudlo, J., Bissbort, C., Glass, A., Grossmann, A., Hauenstein, K., Benecke, R., & Teipel, S. J. (2012). White matter pathology in ALS and lower motor neuron ALS variants: a diffusion tensor imaging study using tract-based spatial statistics. *J Neurol, 259*(9), 1848-1859. doi:10.1007/s00415-012-6420-y

Roche, J. C., Rojas-Garcia, R., Scott, K. M., Scotton, W., Ellis, C. E., Burman, R., . . . Al-Chalabi, A. (2012). A proposed staging system for amyotrophic lateral sclerosis. *Brain, 135*(Pt 3), 847-852. doi:10.1093/brain/awr351

Rose, S., Pannek, K., Bell, C., Baumann, F., Hutchinson, N., Coulthard, A., . . . Henderson, R. (2012). Direct evidence of intra- and interhemispheric corticomotor network degeneration in amyotrophic lateral sclerosis: an automated MRI structural connectivity study. *Neuroimage, 59*(3), 2661-2669. doi:10.1016/j.neuroimage.2011.08.054

Sage, C. A., Van Hecke, W., Peeters, R., Sijbers, J., Robberecht, W., Parizel, P., . . . Sunaert, S. (2009). Quantitative diffusion tensor imaging in amyotrophic lateral sclerosis: revisited. *Hum Brain Mapp, 30*(11), 3657-3675. doi:10.1002/hbm.20794

Trojsi, F., Caiazzo, G., Corbo, D., Piccirillo, G., Cristillo, V., Femiano, C., . . . Tedeschi, G. (2015). Microstructural changes across different clinical milestones of disease in amyotrophic lateral sclerosis. *PloS one, 10*(3), e0119045. doi:10.1371/journal.pone.0119045
